# Supplementary material for: A novel approach to texture recognition combining deep learning orthogonal convolution with regional input features
Source: PeerJ Comput Sci. 2024 Mar 22;10:e1927. doi: 10.7717/peerj-cs.1927 (PMC11041941; doi:10.7717/peerj-cs.1927)
Supplement: Supplemental Information 1 [file peerj-cs-10-1927-s001.docx]

**Table 1. Summary description of OUTEX dataset used.**

| Test Suite ID | Image Type | Texture Class | No.Samples | Information |
| --- | --- | --- | --- | --- |
| Outex_TC_00013 | Color | 68 | 20x68=1360 | Rotation: 00 |
| Outex_TC_00030 | Color | 68 | 9x20x68 = 12240  (9 directions) | Rotations: 00, 05, 10, 15, 30, 45, 60, 75, 90 |
| Outex_TC_00031 | Color | 68 | 40x68=2720 | Rotation: 00  Different resolution |
| Outex_TC_00032 | Color | 68 | 40x68=2720 | Gaussian Noise σ=5 |
| Outex_TC_00033 | Color | 68 | 40x68=2720 | Gaussian Blur ρ=0.5 |
| Outex_TC_00034 | Color | 68 | 40x68=2720 | Different lighting:Inca, Horizon, TL84 |
