# Supplementary material for: A novel approach to texture recognition combining deep learning orthogonal convolution with regional input features
Source: PeerJ Comput Sci. 2024 Mar 22;10:e1927. doi: 10.7717/peerj-cs.1927 (PMC11041941; doi:10.7717/peerj-cs.1927)
Supplement: Supplemental Information 2 — A1- Using normal convolution; A2 –Using separable convolution; A3 –Using separate input paths for RGB and H21. [file peerj-cs-10-1927-s002.docx]

| Label | Input Features | Architecture | Accuracy |
| --- | --- | --- | --- |
| RGB | RGB | A2 | 77.2 |
| 7 | H7 | A1 | 83.0 |
| 7RGB | H7+RGB | A3 | 85.0 |
| 42 | H42 | A1 | 89.3 |
| 21 | H21 | A1 | 89.7 |
| 21S | H21 | A2 | 90.4 |
| 42S | H42 | A2 | 90.4 |
| RGB7S | RGB-H7 | A2 | 92.3 |
| RGB21S-2 | RGB-H21-64 | A2 | 93.0 |
| RGB21S-1 | RGB-H21-16 | A2 | 95.6 |

Table 2. Architecture Description: A1- Using normal convolution; A2 – Using separable convolution; A3 – Using separate input paths for RGB and H21.
