# Supplementary material for: A novel approach to texture recognition combining deep learning orthogonal convolution with regional input features
Source: PeerJ Comput Sci. 2024 Mar 22;10:e1927. doi: 10.7717/peerj-cs.1927 (PMC11041941; doi:10.7717/peerj-cs.1927)
Supplement: Supplemental Information 3 [file peerj-cs-10-1927-s003.docx]

| Dataset | ACC | SAMPLE SIZE | CLASS | Description |
| --- | --- | --- | --- | --- |
| OUTEX_TC_00013 | 95.6 | 1360 Train:Test split 30% | 68 | Coloured version |
| OUTEX_TC_00031 | 93.7 | 2720 Train:Test split 30% | 68 | Different Resolution |
| OUTEX_TC_00032 | 95.2 | 2720 Train:Test split 30% | 68 | Gaussian Noise |
| OUTEX_TC_00033 | 95.2 | 2720 Train:Test split 30% | 68 | Gaussian Blurred |
| OUTEX_TC_00034 | 93 | 2720 Train:Test split 30% | 68 | Different illuminants |

Table 3. Results on various OUTEX datasets
