# Supplementary material for: A novel approach to texture recognition combining deep learning orthogonal convolution with regional input features
Source: PeerJ Comput Sci. 2024 Mar 22;10:e1927. doi: 10.7717/peerj-cs.1927 (PMC11041941; doi:10.7717/peerj-cs.1927)
Supplement: Supplemental Information 4 [file peerj-cs-10-1927-s004.docx]

| **Dataset** | **ACC** | **SAMPLE SIZE** | **CLASS** | **Remarks** |
| --- | --- | --- | --- | --- |
| OUTEX_TC_00030-1 | 90.9 | Train: 1360  Test: 5 | 68 | trained 00 only; tested on 05 direction |
| OUTEX_TC_00030-2 | 87.1 | Train: 1360  Test: 5 | 68 | trained 00 only; tested on 10 direction |
| OUTEX_TC_00030-3 | 87.3 | Train: 1360  Test: 5 | 68 | trained 00 only; tested on 15 direction |
| OUTEX_TC_00030-4 | 86.8 | Train: 1360  Test: 5 | 68 | trained 00 only; tested on 30 direction |
| OUTEX_TC_00030-5 | 85.9 | Train: 1360  Test: 5 | 68 | trained 00 only; tested on 45 direction |
| OUTEX_TC_00030-6 | 84.4 | Train: 1360  Test: 5 | 68 | trained 00 only; tested on 60 direction |
| OUTEX_TC_00030-7 | 85.6 | Train: 1360  Test: 5 | 68 | trained 00 only; tested on 75 direction |
| OUTEX_TC_00030-8 | 92.6 | Train: 1360  Test: 5 | 68 | trained 00 only; tested on 90 direction |

Table 4. Experiment 4: Results on the OUTEX rotated dataset
