# Supplementary material for: A novel approach to texture recognition combining deep learning orthogonal convolution with regional input features
Source: PeerJ Comput Sci. 2024 Mar 22;10:e1927. doi: 10.7717/peerj-cs.1927 (PMC11041941; doi:10.7717/peerj-cs.1927)
Supplement: Supplemental Information 5 [file peerj-cs-10-1927-s005.docx]

| Database | Our | GoogleNet | ResNet | VggNet | AlexNet |
| --- | --- | --- | --- | --- | --- |
| OUTEX_TC_00013 | **95.6** | 79.1 | 84.3 | 84.0 | 80.4 |
| OUTEX_TC_00030 | **90.9** | 88.2 | 92.7 | 91.8 | 90.0 |
| OUTEX_TC_00031 | **93.7** | 80.6 | 86.1 | 85.8 | 82.8 |
| OUTEX_TC_00032 | **95.2** | 82.6 | 85.7 | 85.9 | 91.5 |
| OUTEX_TC_00033 | **95.2** | NA | NA | NA | NA |
| OUTEX_TC_00034 | **93.0** | 80.5 | 83.9 | 83.9 | 82.6 |

Table 5. Comparison of our results with various other deep learning architecture on various OUTEX dataset
