# Supplementary figures and images for: A novel approach to texture recognition combining deep learning orthogonal convolution with regional input features
Source: PeerJ Comput Sci. 2024 Mar 22;10:e1927. doi: 10.7717/peerj-cs.1927 (PMC11041941; doi:10.7717/peerj-cs.1927)

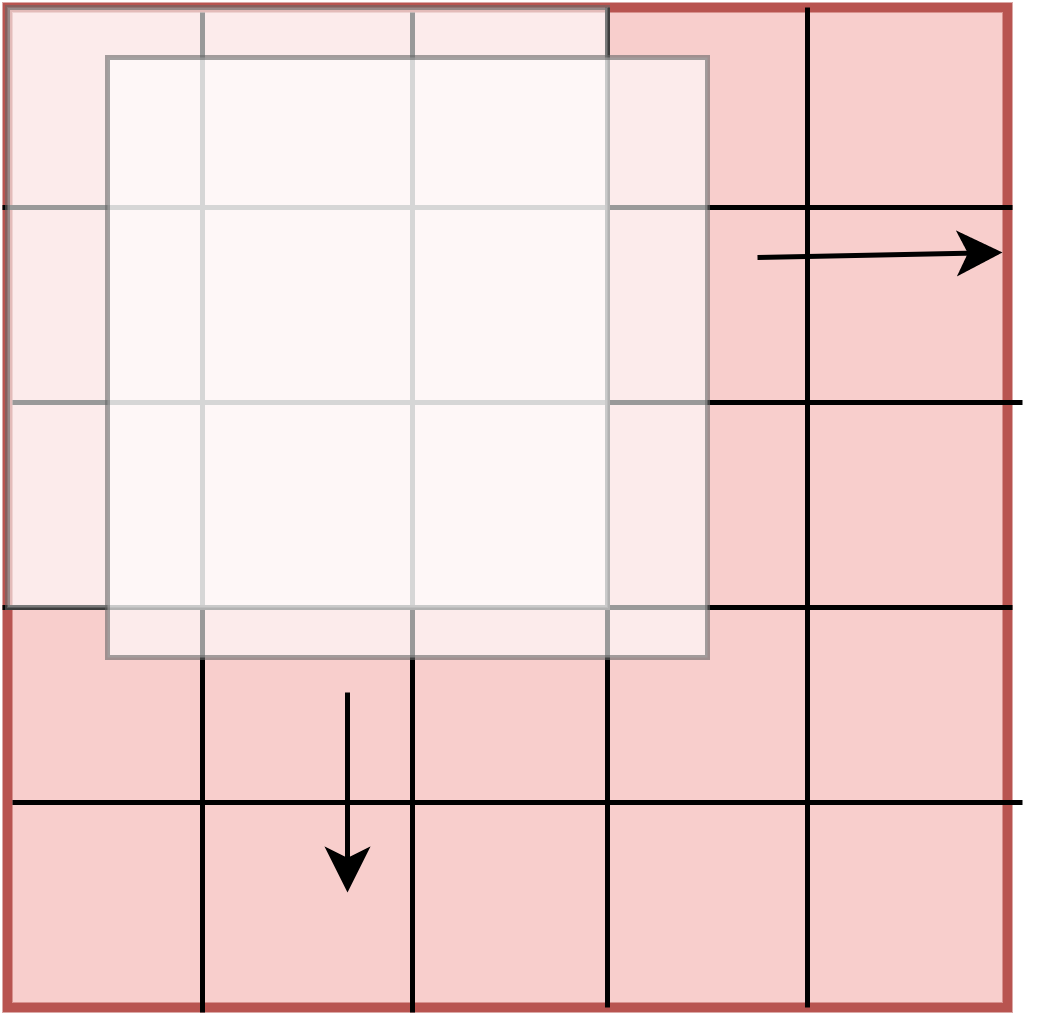

Supplement: Supplemental Information 6 [file peerj-cs-10-1927-s006.png]

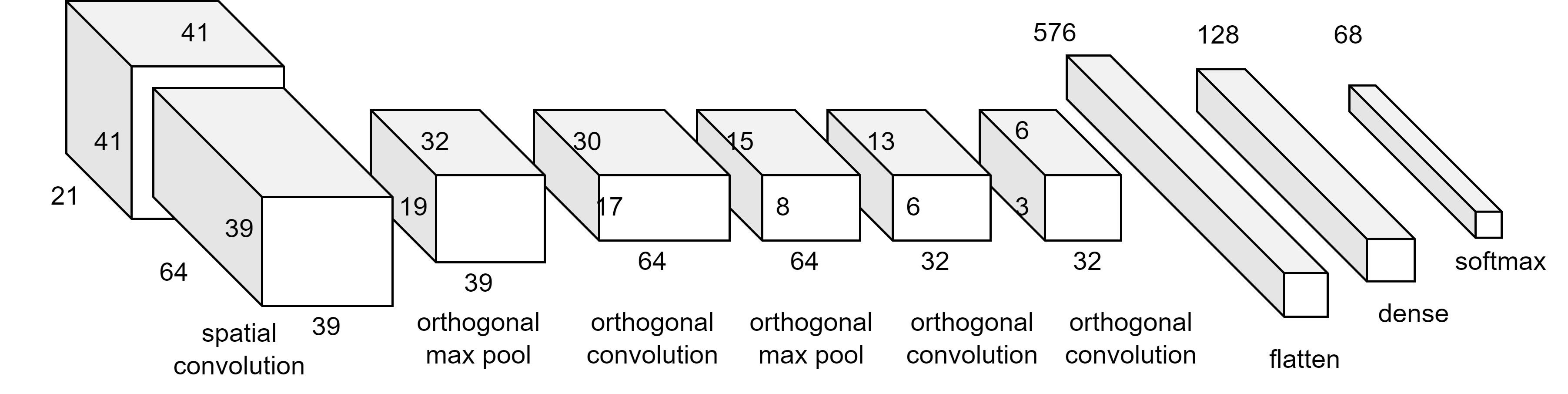

Supplement: Supplemental Information 7 [file peerj-cs-10-1927-s007.png]

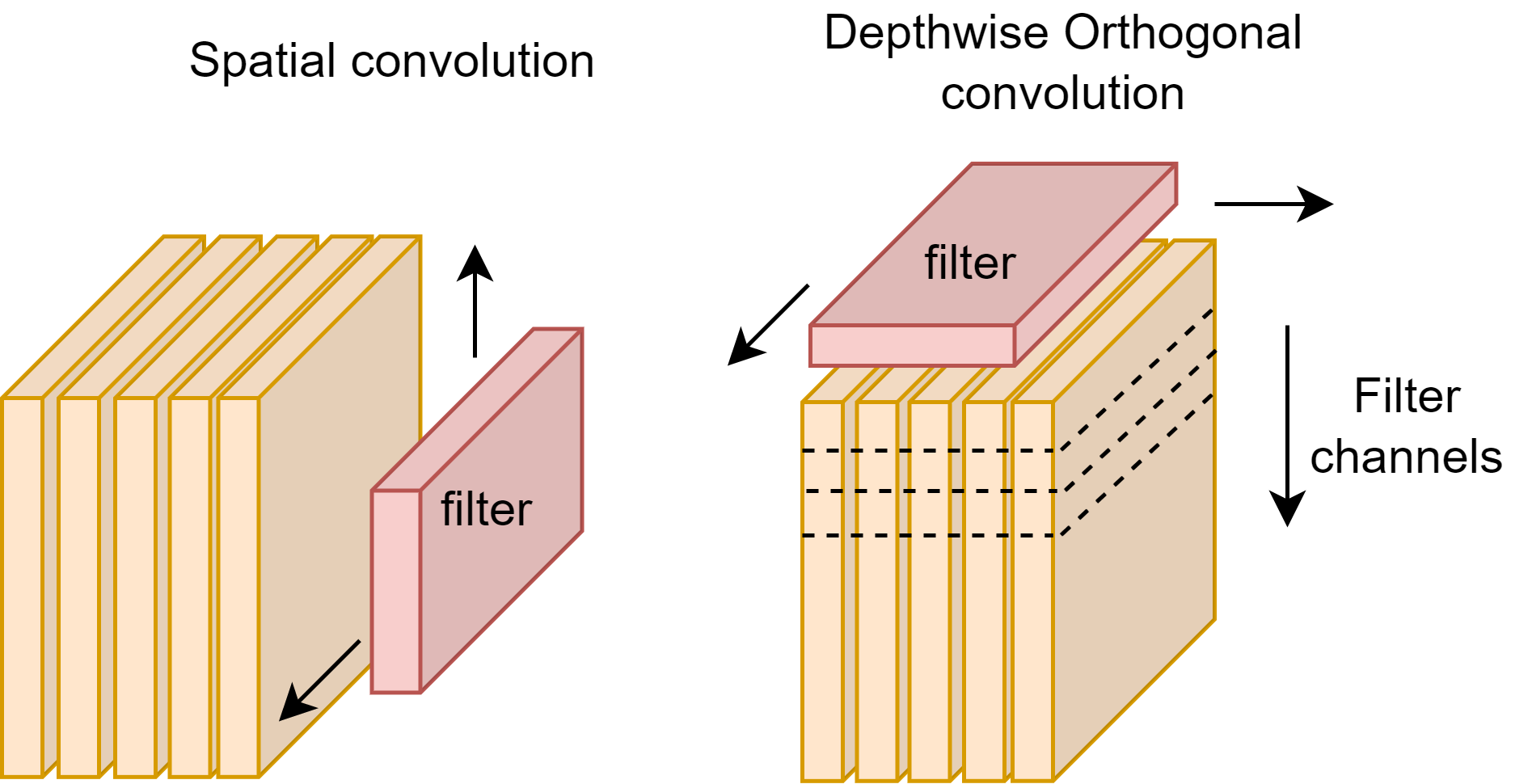

Supplement: Supplemental Information 8 [file peerj-cs-10-1927-s008.png]

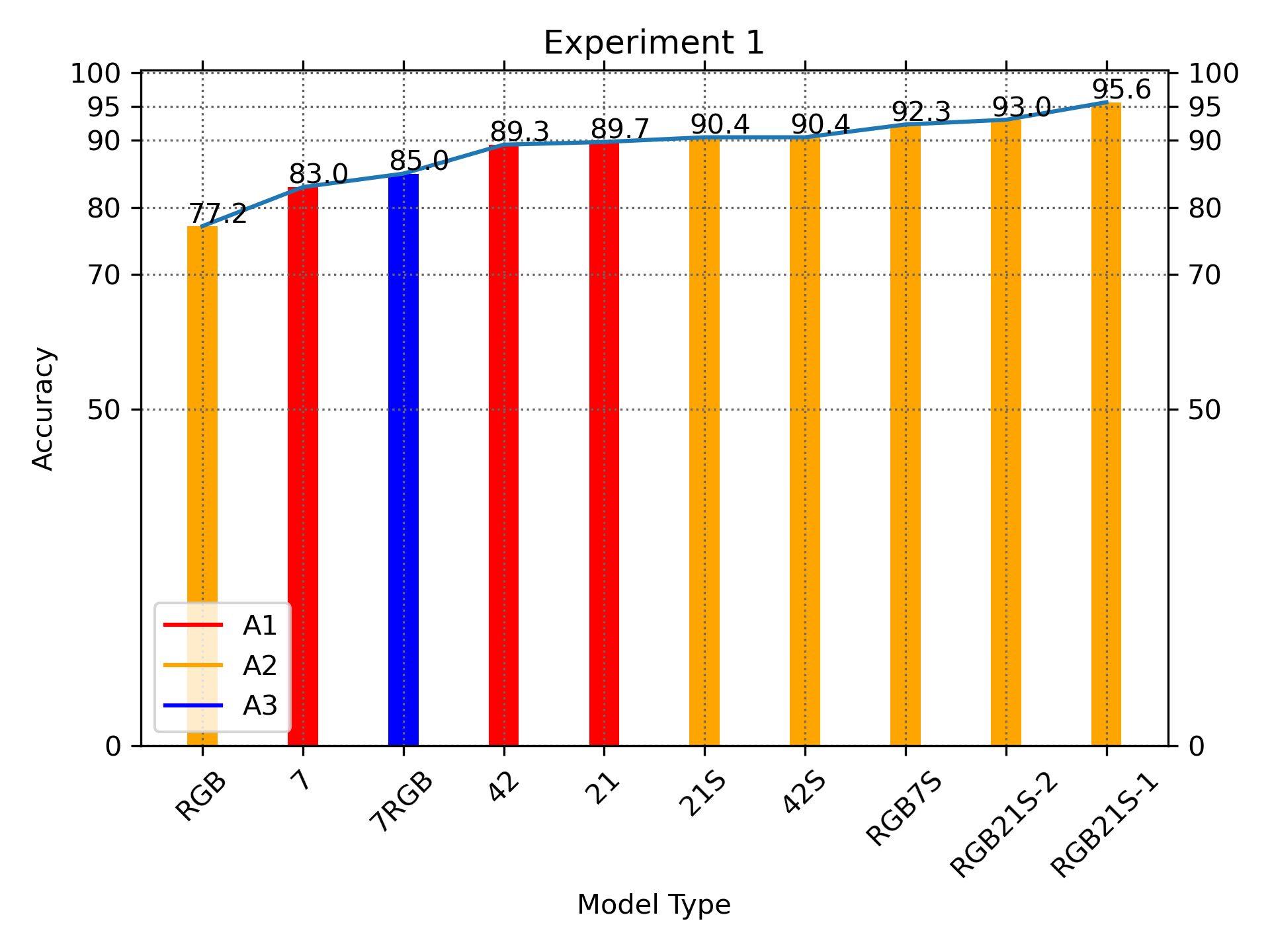

Supplement: Supplemental Information 9 [file peerj-cs-10-1927-s009.png]

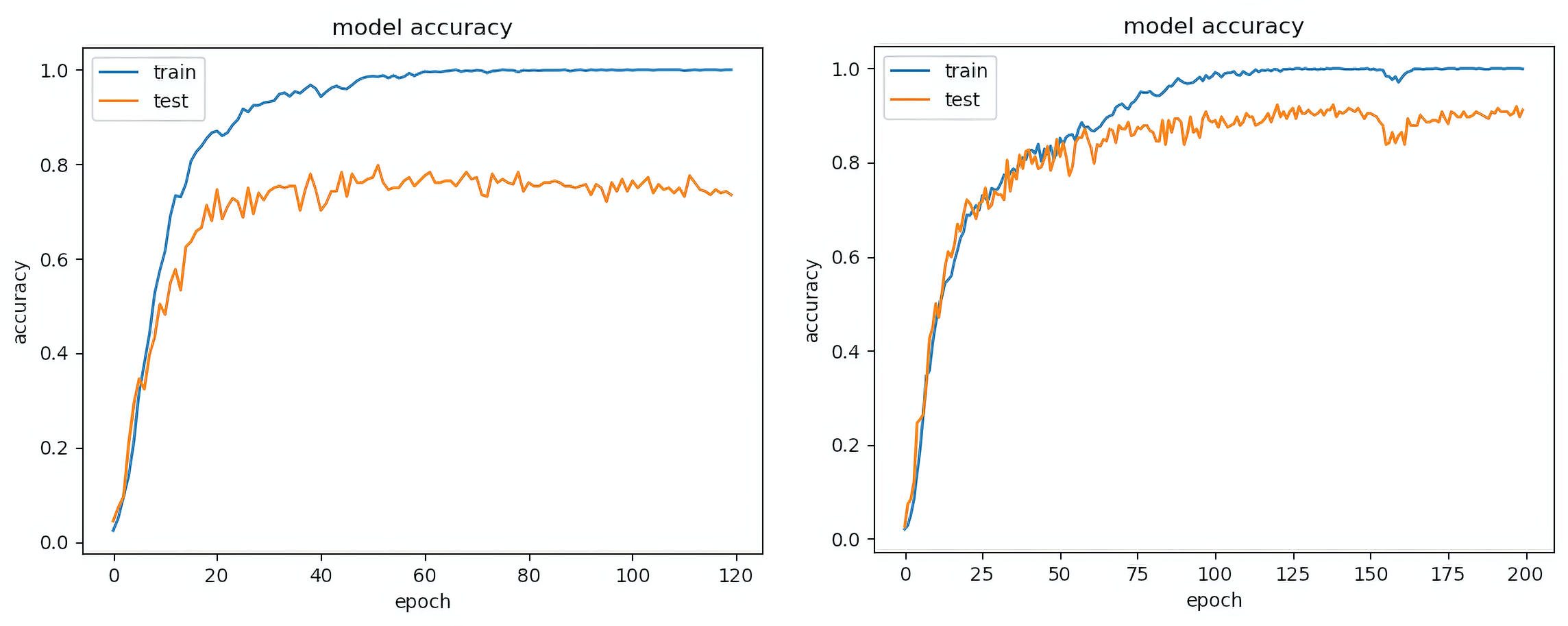

Supplement: Supplemental Information 10 [file peerj-cs-10-1927-s010.png]

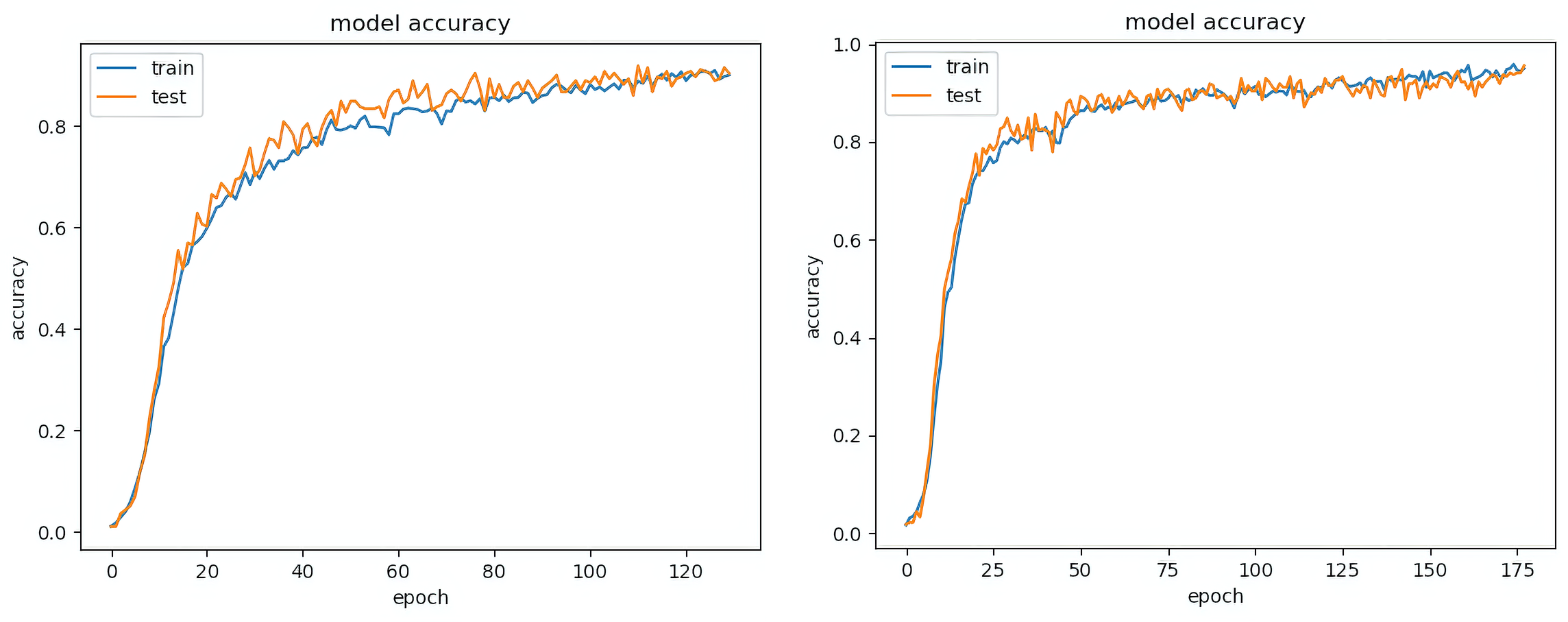

Supplement: Supplemental Information 11 [file peerj-cs-10-1927-s011.png]

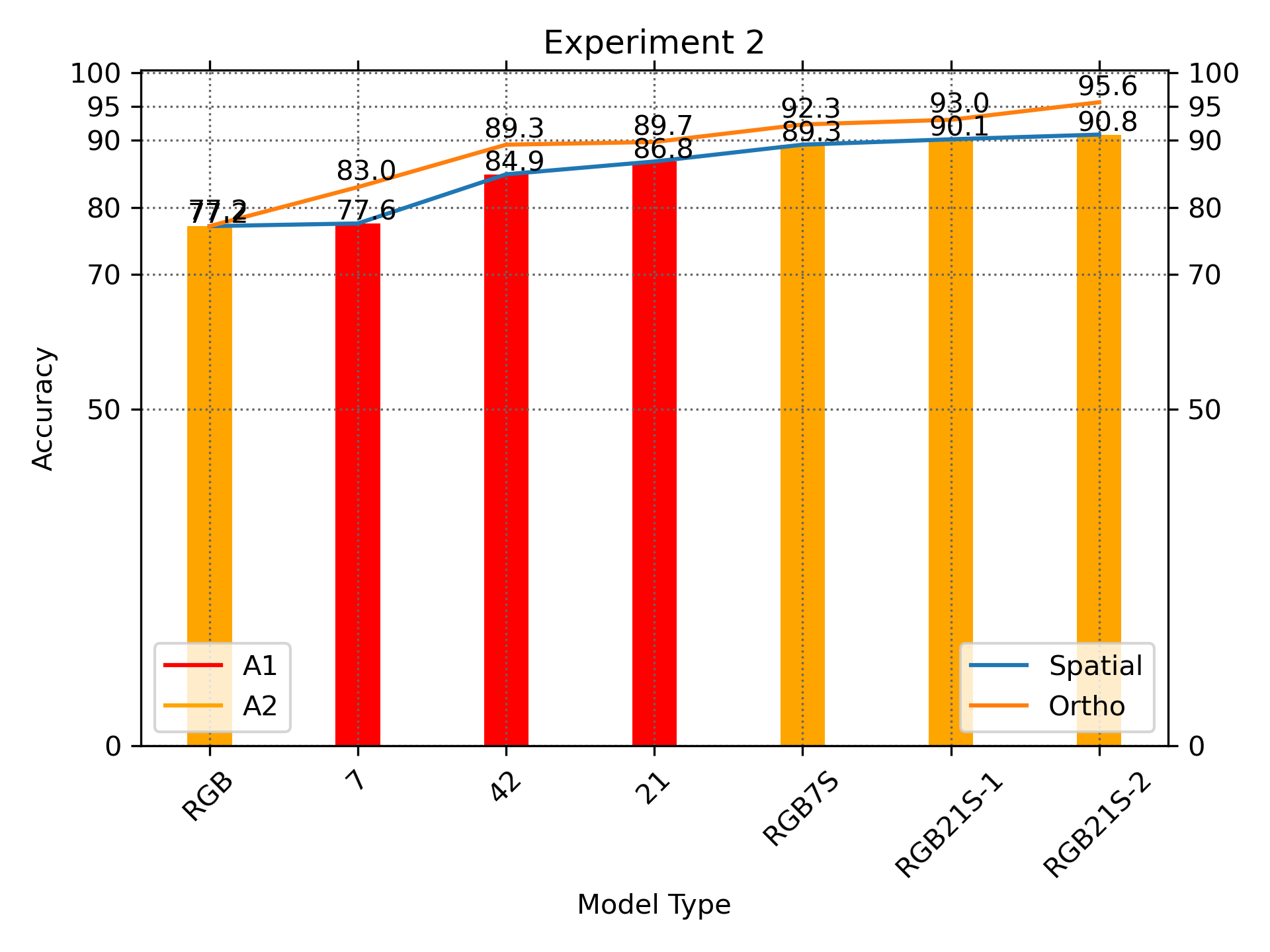

Supplement: Supplemental Information 12 [file peerj-cs-10-1927-s012.png]

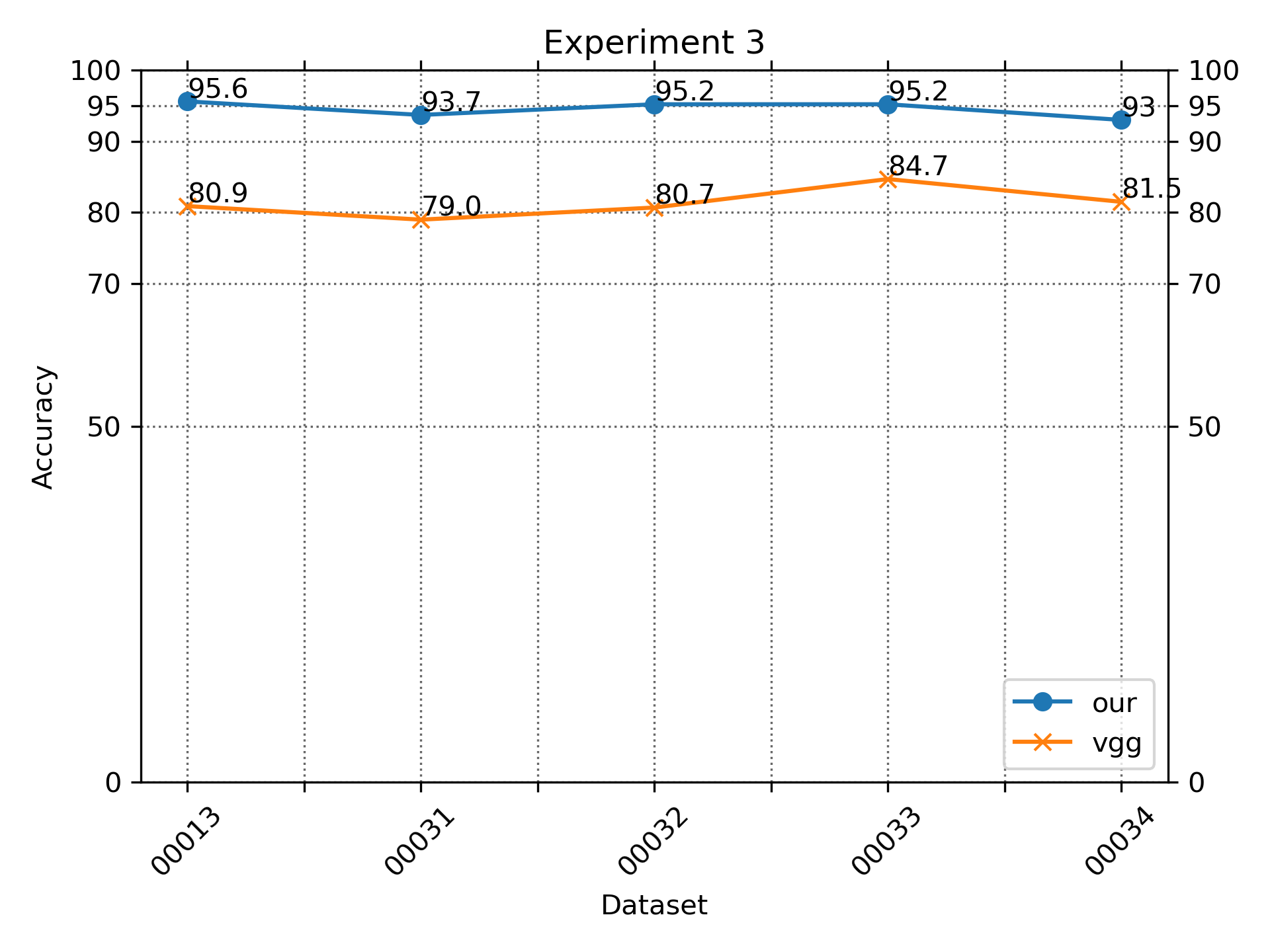

Supplement: Supplemental Information 13 [file peerj-cs-10-1927-s013.png]

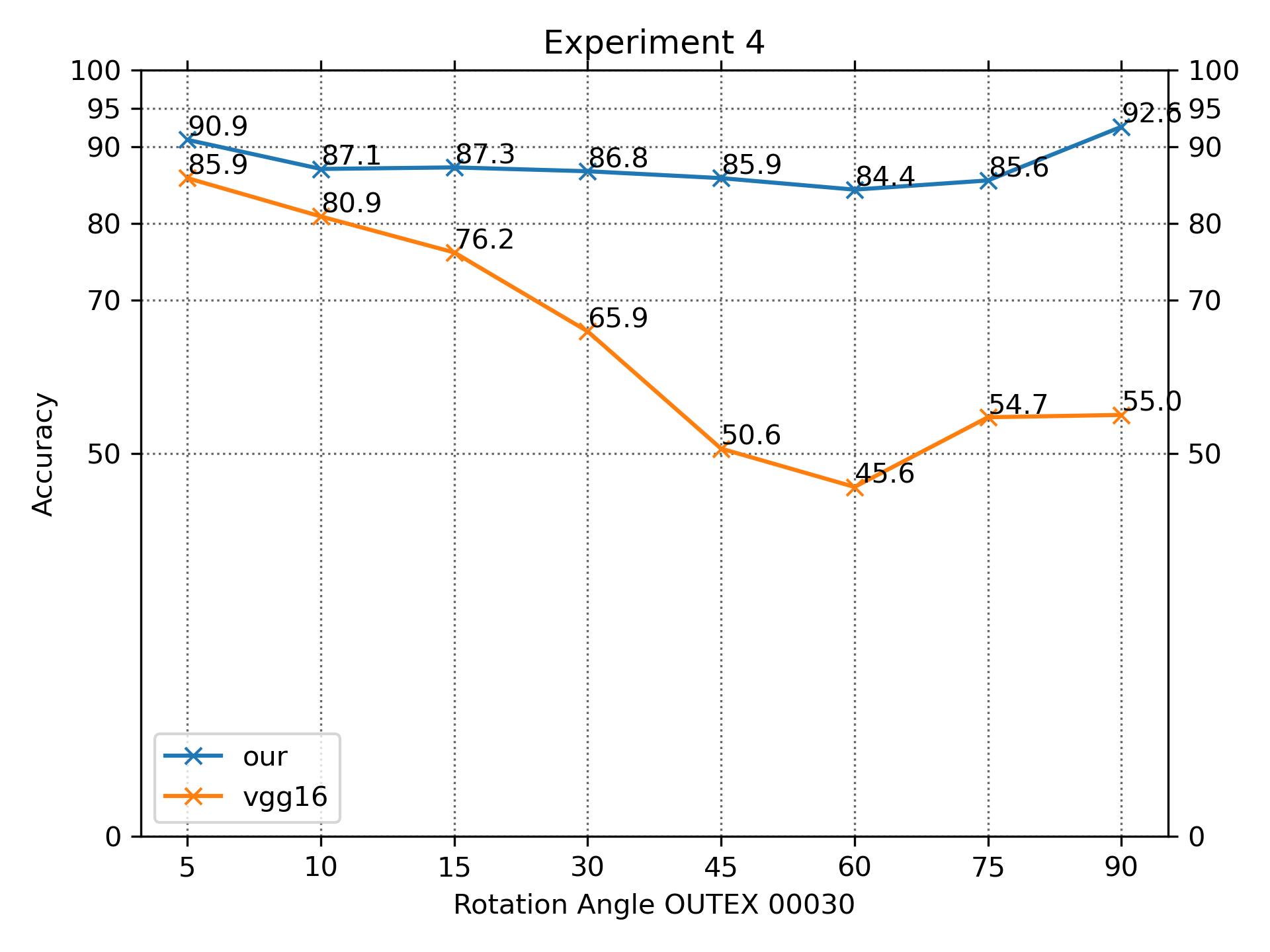

Supplement: Supplemental Information 14 [file peerj-cs-10-1927-s014.png]

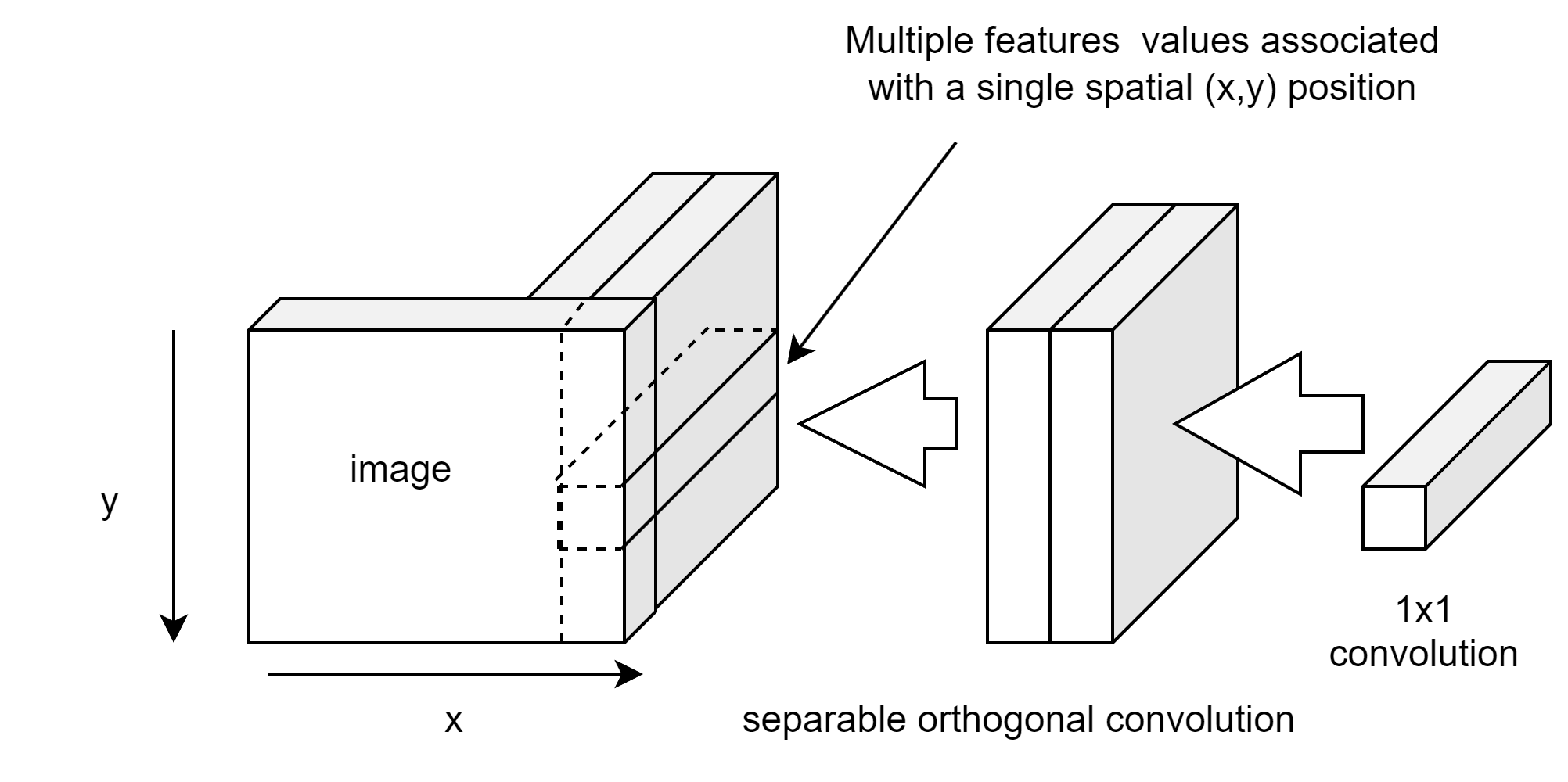

Supplement: Supplemental Information 15 [file peerj-cs-10-1927-s015.png]

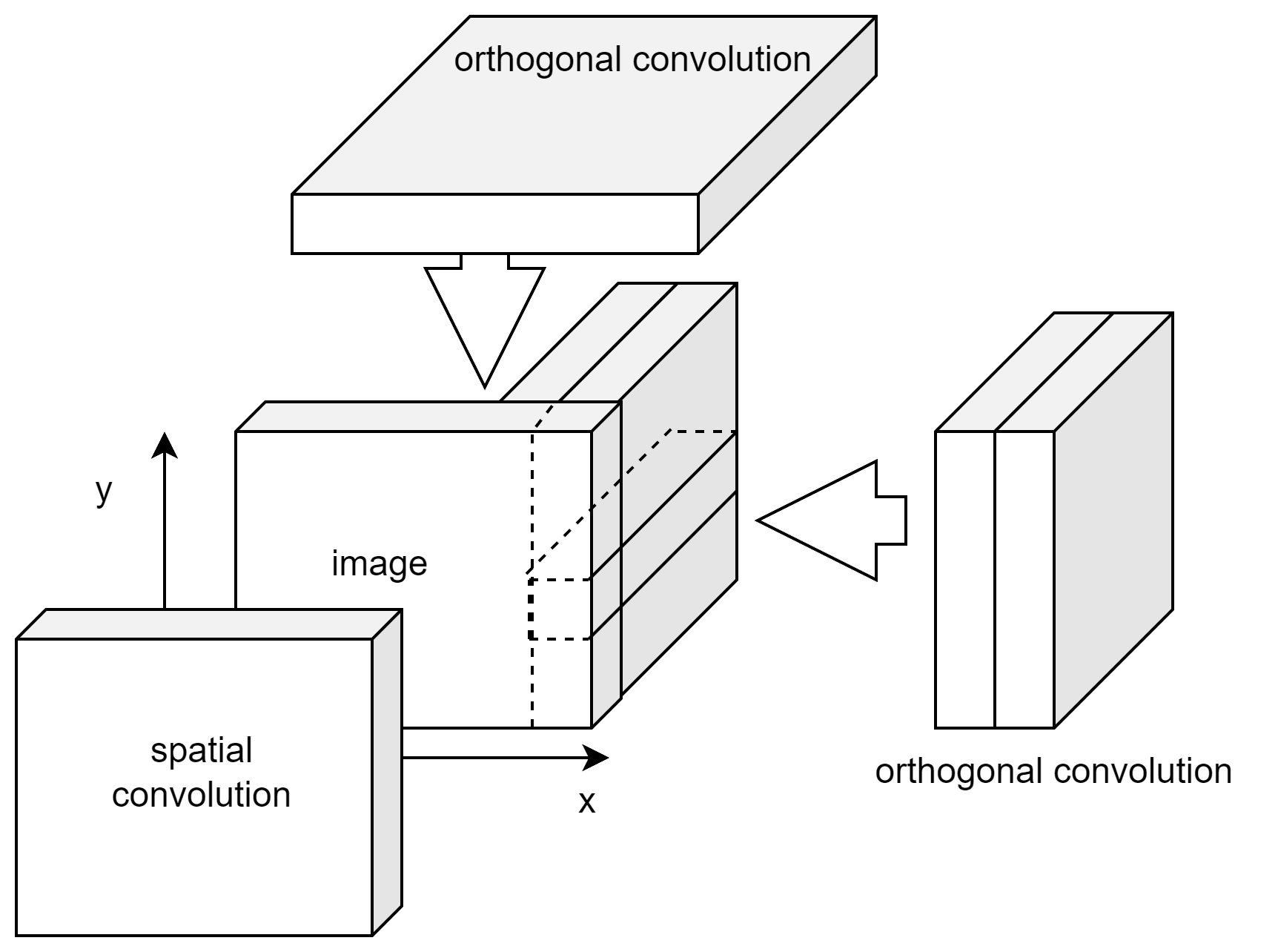

Supplement: Supplemental Information 16 [file peerj-cs-10-1927-s016.png]
